# Small Bowel Tumors: A 7-Year Study in a Tertiary Care Hospital

**DOI:** 10.3390/cancers17091465

**Published:** 2025-04-27

**Authors:** Sergiu Marian Cazacu, Dan Cârțu, Mihai Popescu, Liliana Streba, Bogdan Silviu Ungureanu, Vlad Florin Iovănescu, Mihai Cimpoeru, Cecil Sorin Mirea, Valeriu Marian Surlin, Stelian Mogoantă, Mirela Marinela Florescu

**Affiliations:** 1 Gastroenterology Department, University of Medicine and Pharmacy, 200349 Craiova, Romania; sergiu.cazacu@umfcv.ro (S.M.C.); bogdan.ungureanu@umfcv.ro (B.S.U.); vlad.iovanescu@umfcv.ro (V.F.I.); 2 Surgery Department, University of Medicine and Pharmacy, 200349 Craiova, Romania; dan.cartu@umfcv.ro (D.C.); cecil.mirea@umfcv.ro (C.S.M.); valeriu.surlin@umfcv.ro (V.M.S.); stelian.mogoanta@umfcv.ro (S.M.); 3 Imaging Department, University of Medicine and Pharmacy, 200349 Craiova, Romania; mihai.popescu@umfcv.ro; 4 Oncology Department, University of Medicine and Pharmacy, 200349 Craiova, Romania; 5 Gastroenterology Department, Clinical Emergency Hospital, Tabaci Street No. 1, 200642 Craiova, Romania; mcimpoeru810@gmail.com; 6 Pathology Department, University of Medicine and Pharmacy, 200349 Craiova, Romania; mirela.florescu@umfcv.ro

**Keywords:** small bowel tumors, adenocarcinoma, squamous carcinoma, gastrointestinal endoscopy, surgery

## Abstract

(1) Background: Tumors of the small bowel represent 3–6% of gastrointestinal neoplasms and 3–6% of GI malignancies. The difficulties regarding the diagnosis are associated with larger tumors at the moment of the diagnosis and with advanced forms of malignant tumors, associated with a dismal prognosis. (2) Methods: We performed an observational, retrospective, cohort study that included patients with small bowel tumors admitted to the Craiova County Emergency Clinic Hospital between 1 January 2017 and 31 December 2023. The data were collected from the analysis of the patient’s discharge documents from the Hippocrates computer system of the hospital and the evaluation of endoscopy databases. Patients under 16 years of age, those with no pathological confirmation of the malignancy, and those with insufficient data were excluded. (3) Results: A total of 80 cases of small bowel tumors were diagnosed; 72.5% were malignant, of which 10.3% were metastases. The most frequent primary malignant small bowel tumor was adenocarcinoma; two squamous cell carcinomas were noted. CT scans and upper digestive endoscopy represent the most frequent imaging methods for the diagnosis. The prognosis for malignant tumors was poor, with a 41% 5-year survival rate. (4) Conclusions: Small bowel tumors are rarely encountered, with 72.5% being malignant, and were diagnosed at large dimensions and in advanced stages for malignant tumors, with a dismal prognosis.

## Simple Summary

Tumors of the small bowel are rarely encountered and difficult to diagnose; the symptoms are nonspecific until the late stages. The introduction of videocapsule endoscopy and balloon- or spiral-assisted enteroscopy has improved the diagnosis, although many large tumors are still diagnosed by CT scan and in emergency settings. The most frequent malignant small bowel tumors are adenocarcinoma, lymphoma, GIST, and neuroendocrine tumors. We present a 7-year study of 80 small bowel tumors admitted to our clinic, of which 62.2% were primary malignant tumors and 10.3% were metastases. Adenocarcinoma represents the most frequent malignant tumor; two squamous carcinomas were diagnosed. The 5-year survival rate was 41%.

## 1. Introduction

Tumors of the small bowel are relatively rare forms, accounting for 3–6% of gastrointestinal (GI) neoplasms [1,2,3,4]. An increased prevalence was noted in the last several decades, possibly associated with the progress in diagnostic procedures [5].

Several pathological types have been described in small bowel tumors (SBTs) [6]. Malignant tumors are more frequent [2] and include carcinomas (most frequently adenocarcinomas and rarely squamous cell carcinomas), neuroendocrine tumors, lymphomas, and sarcomas; intestinal stromal tumors and mesenchymal tumors are also often described [1,3,5,7,8,9,10,11]. Rare cases of primary squamous carcinomas [12,13,14,15,16,17,18,19,20,21,22,23] and leiomyosarcomas [24] have been noted. Metastatic small bowel tumors originating from colon, prostate, breast, and lung cancers, or from skin melanoma, have also been recorded [1,3,7,13].

The symptoms of patients with SBT are variable and sometimes correlated with the pathological type [7]. The most frequent complaints are nausea and/or vomiting, abdominal pain, bleeding, and weight loss [1,5,25,26]. Bleeding may be either overt (melena) or occult (anemia). Bowel obstruction (by the tumor or by intussusception), or sometimes perforations, are seen frequently [3,4,27,28,29]. This can sometimes even occur in benign SBT [30,31,32,33,34]. Emergency surgery may be needed; one study from Turkey found a rate of 17.6% for emergency procedures [5]. SBT represents the second cause of obscure gastrointestinal bleeding [4], and in various studies of patients with iron-deficient anemia, the rate of SBT after negative bidirectional endoscopy was reported in up to 8.9% of cases [3], with a pooled frequency of 1.25% in a systematic review [35]. Jejunal tumors are associated more frequently with bleeding, and ileal tumors with abdominal pain [2]. Nonspecific symptoms are usually associated with a six- to nine-month delay in diagnosis [1].

Survival rates for malignant SBT are hard to estimate because of the high variability of the tumor types. Some models involving Deep Learning or other types of artificial intelligence were studied [36].

The diagnosis can be difficult, especially in the mid-segment of the small bowel. Duodenal and sometimes jejunal tumors can be diagnosed by upper digestive endoscopy, while ileo-colonoscopy can be used for SBT in the terminal ileum. Enteroscopy (spiral, single-, or double-balloon enteroscopy) is very useful [4,37,38,39,40,41,42,43,44,45,46,47,48,49], as well as videocapsule enteroscopy (VCE). Anterograde combined with retrograde balloon enteroscopy can assess 70% of the small bowel [4], while motorized spiral enteroscopy can frequently evaluate the entire small bowel, sometimes by using only the anterograde (oral) route [4]. VCE introduction has revolutionized small bowel pathology diagnosis and foremost gastrointestinal bleeding with unclear etiology [50], as it is easy to perform, non-invasive, and does not need sedation. However, the rate of entire small bowel examinations ranges between 67 and 76.6% [51,52,53], and it cannot perform biopsies or therapeutic maneuvers [3]. Some lesions (especially gastrointestinal stromal tumors—GISTs, and tumors located in the duodenum or Treitz area) may be missed [48,54]. In two studies of 79 and 16 patients with SBT, 16.7% were missed by VCE [55,56]. Even large subepithelial lesions as large as 3 cm can be missed [57]. Capsule retention represents another disadvantage; even if rates of 3% are usually seen in normal investigations [53], in SBT, the retention rates go up to 10 or even 20% [54]. A meta-analysis comparing enteroscopy with VCE found a good sensitivity and specificity for enteroscopy (89% and 97%, respectively) [58]. The main advantage of enteroscopy is represented by the possibility of both biopsies and tattooing the lesions, which can help the surgeons to find the lesion [4]. Transabdominal ultrasound can sometimes visualize the tumor, but the sensitivity is only 50.5%, with 100% specificity [59]; in tumors above 2 cm in diameter, with circumferential extension or ulcerative pattern, the sensitivity increases above 90% [59]. Computed tomography (CT) scans or magnetic resonance imaging (MRI) have a higher accuracy; however, small tumors can be easily missed [1]. In a study of 79 SBTs, including 9 missed by VCE, the accuracy for CT scans was 55.8%, and for small bowel follow-through was 46.1% [55]. In another study of 16 neuroendocrine tumors, the detection rate for CT scans was 62.5% [56]. The use of CT or MRI enteroclysis [1,60] significantly increased the accuracy of SBT detection. Digital angiography can be useful in small SBTs with active bleeding [61].

Because subepithelial lesions of the small bowel tract can be missed on VCE examination, a score named SPICE (Smooth, Protruding Lesion Index of Capsule Endoscopy) was proposed, which includes four criteria: mass with ill-defined boundaries, with a diameter larger than its height, non-visible lumen, and mass image visible for less than 10 min [4,48]. Another score proposed for the probability of a mass lesion at VCE examination includes bleeding, mucosal disruption, irregular surface, white villi, and color [62]; a score of four or more was associated with a high probability of a small bowel mass lesions, while a score of two or less was associated with a low likelihood [62]. Artificial intelligence may aid VCE accuracy for SBT detection [63,64], with a significantly reduced time for examination (average 4.5 min), a sensitivity as high as 98–99%, and an error in diagnosis below 3% [64].

Our study was designed to fill the existing literature gaps regarding small bowel tumors in Romania by assessing the prevalence, diagnosis, pathological types, and prognosis of benign and malignant small bowel tumors in patients admitted between January 2017 and December 2023 in a tertiary care hospital. Several studies in Eastern and Southern Europe have assessed the prevalence of small bowel tumors [5,10,29,37,65,66,67], but some included only small series [66], only surgical cases [10,29,65,66,67], or were limited to malignant tumors [5,10,66]. Given the paucity of data regarding small bowel tumors in Eastern and Southern Europe, a study presenting an analysis of both benign and malignant small bowel tumors in Romania helps to describe the epidemiology of SBT, and by including cases without surgical management, we can better estimate survival analysis in malignant small bowel tumors.

## 2. Materials and Methods

### 2.1. Patient Selection

We conducted an observational, retrospective, cohort study that included patients with small bowel tumors admitted to the Craiova County Emergency Clinic Hospital between 1 January 2017 and 31 December 2023. Data were collected from the Hippocrates (Romanian Soft Company, Bucharest, Romania) digital registration system of the hospital with diagnostic codes of definite or possible small bowel tumors (C17.0—malignant duodenal tumor; C17.1—malignant jejunal tumor; C17.2—malignant ileal tumor; C17.8—malignant tumor surpassing small bowel; C17.9—malignant small bowel tumors, unprecise; D01.7—carcinoma in situ of other specified digestive organs; D13.3—benign small bowel tumors; D37.2—small bowel tumors with unspecified and unknown evolution; D37.7 and D37.9—other digestive tumors with unspecified and unknown evolution), as well as K56 (paralytic ileus and intestinal obstruction without hernia), and were supplemented as needed by analyzing the patient medical records. Patients under 16 years of age, with no pathological confirmation of the malignancy, or incomplete records were excluded.

The diagnosis of small bowel tumors was based on clinical data; digestive endoscopy—upper digestive endoscopy, push enteroscopy, or ileo-colonoscopy performed exclusively on an Olympus Exera III system (Olympus Europe, Hamburg, Germany) and videocapsule endoscopy (Olympus EndoCapsule, Olympus Europe, Hamburg, Germany); and imaging—CT scan (Siemens Somatom go. TOP 128 slice reconstructed, Siemens Healthineers, Erlangen, Germany), MR imaging (Siemens—Magnetom Symphony 1,5T, Siemens Healthineers, Erlangen, Germany), or incidentally during abdominal surgery. It was then confirmed by pathology examination of endoscopically resected tumors (performed in the Gastroenterology Department) or surgically resected specimens, or by intraoperative biopsy in one of the three surgery departments of the hospital (in case of inoperable lesions). Only small bowel tumors with confirmed histopathology were included. The symptoms, along with the presence of complications, were recorded. The CT scan and MR imaging noted the dimensions of the lesion (if the lesion was seen), the parietal thickening, the retrograde dilation of the small bowel or the stomach, and the presence or absence of invaded lymph nodes, as well as distant metastases.

The diagnosis was confirmed in all patients by histopathological exam (with detailed information related to the benign or malignant type of tumor and also to the epithelial or mesenchymal origin, grading, lymphovascular or perineural invasion, and pTNM classification in malignant tumors); immunohistochemical evaluation was performed in a subgroup of the patients. The tumor stage was evaluated using the 8th edition of the “TNM Malignant Tumor Classification” from the International Cancer Control Union [68]. The immunohistochemical panel applied includes markers for squamous cell carcinoma, malignant melanoma (Melan A, HIMB45), the evaluation of endothelial differentiation (CD31, CD34), or the tumor proliferation index Ki-67. Final TNM staging, grading, and vascular and perineural invasion were also analyzed.

This study was approved by the Clinical County Emergency Hospital of Craiova Ethics Committee (Approval Number 17886/24 April 2024) on the following information: (1) data were collected within a retrospective, observational study; (2) the study did not interfere with current medical care; (3) data were collected and analyzed anonymously so that the patient data confidentiality would not be breached.

### 2.2. Data Collection and Statistical Analysis

The extracted data were processed in Microsoft Excel (Microsoft Corp., Redmond, WA, USA), with the XLSTAT 2016 add-on for MS Excel (Addinsoft SARL, Paris, France). The frequencies are presented as absolute numbers of cases and percentages. Survival analysis was performed using R software (version 4.3) with the survival and survminer packages. Survival analysis was performed using the Kaplan–Meier method to estimate survival probabilities, and differences between groups were assessed with the log-rank test. Cox proportional hazards regression models were used to evaluate the association between potential risk factors and survival outcomes. The multivariate Cox model included variables with a *p*-value < 0.05 in univariate analysis or clinical relevance. Hazard ratios (HRs) with 95% confidence intervals (CIs) are reported to quantify effect sizes.

## 3. Results

### 3.1. Main Characteristics of Patients

**Figure 1:**
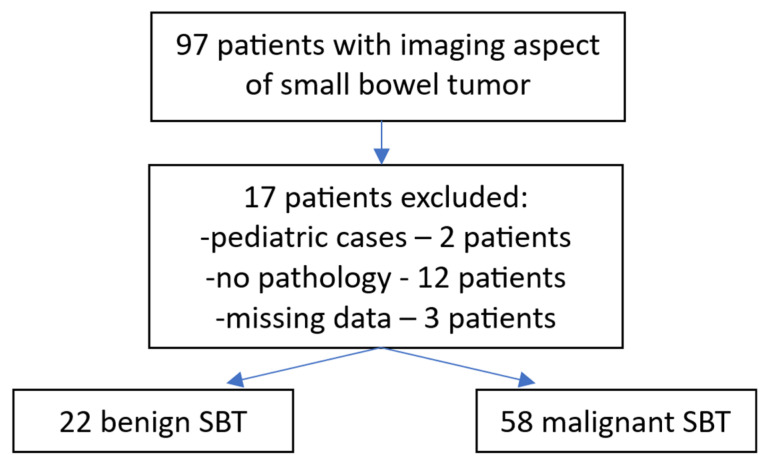
A diagram showing the inclusion of patients and tumor types.

During the study period, 80 cases of small bowel tumors were admitted; 58 were malignant tumors and 22 were benign tumors (Figure 1). Mean age was similar for benign and malignant tumors (63.1 for benign and 64 for malignant tumors, *p* = 0.7614); male gender was predominant for both benign and malignant SBT. There was an almost equal distribution to the duodenum, jejunum, and ileum; five cases presented multiple tumors located in the small bowel.

The most frequent symptoms at admission were abdominal pain and nausea with or without vomiting; complications were noted in 46.2% of cases (intestinal occlusion, bleeding, invagination, and perforation). In-hospital death was recorded in 10 cases (12.5%), being related to complications and advanced malignant diseases (Table 1).

**Figure 2:**
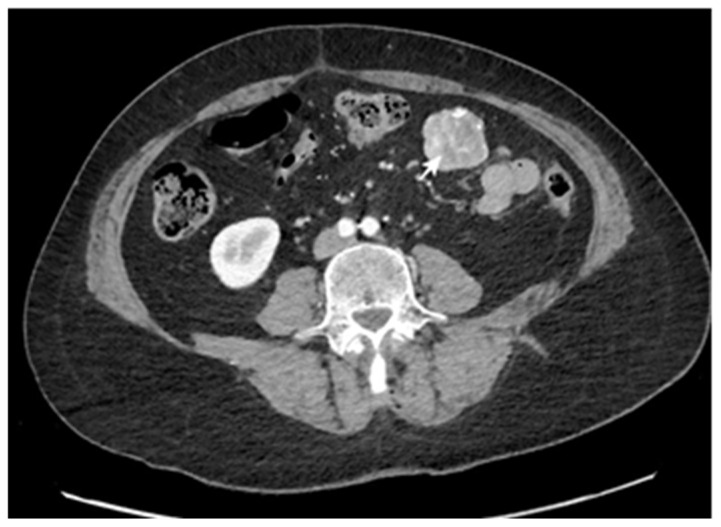
CT scan post-contrast with heterogeneous uptake and central necrosis, vascular peripheral enhancement, and regular contour, with no loco-regional extension. The pathology exam revealed an intestinal stromal tumor.

**Figure 3:**
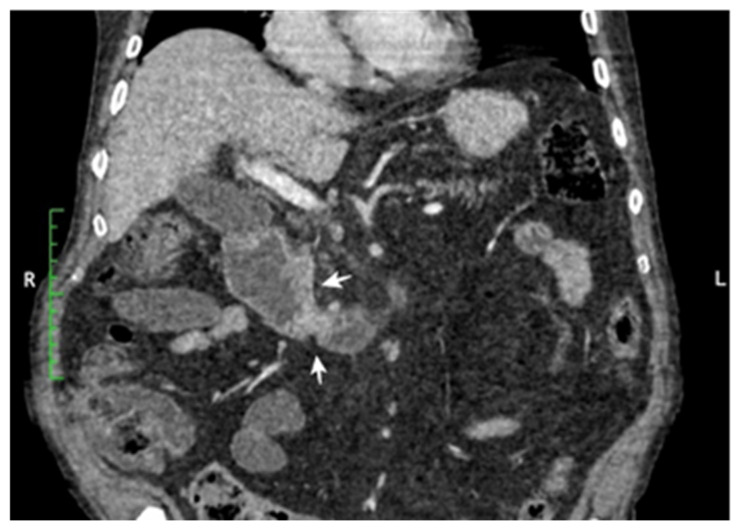
Coronal CT reconstruction post-contrast: infiltrative tumor located on the upper part of the small bowel, with circumferential distribution. Small lymph nodes are visible. Pathology revealed duodenal adenocarcinoma.

**Figure 4:**
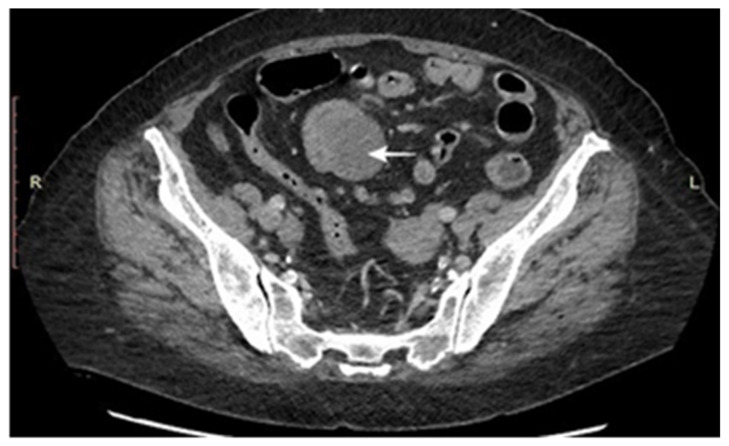
CT scan: tumoral mass with ileal location and predominant extraluminal extension, ileal luminal stenosis and upstream dilation of the small bowel, moderate enhancement, and mesenteric fat infiltration. Several mesenteric lymph nodes up to 1.4 cm.

**Figure 5:**
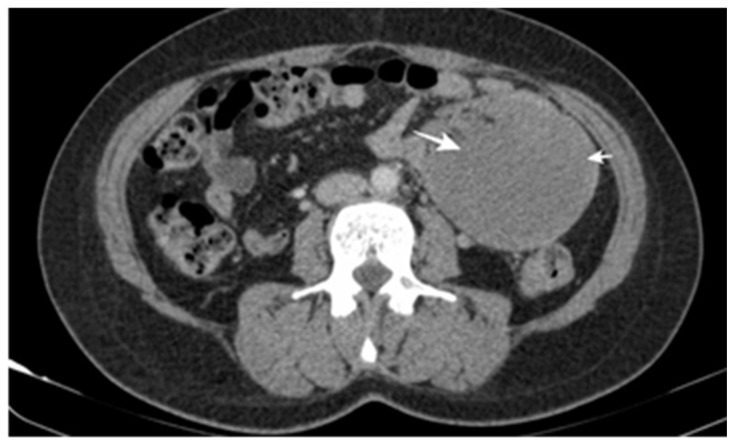
CT post-contrast axial scan: heterogeneous mass at the level of jejunal loops, round-shaped, with regular contours. The pathology exam revealed a mesenchymal angiomatous tumor.

**Figure 6:**
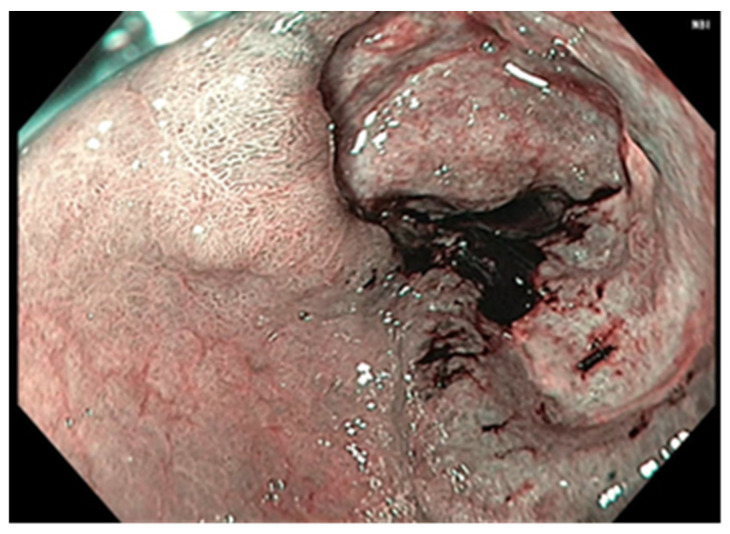
Upper digestive endoscopy: duodenal carcinoma visible through the dilated pyloric orifice.

**Figure 7:**
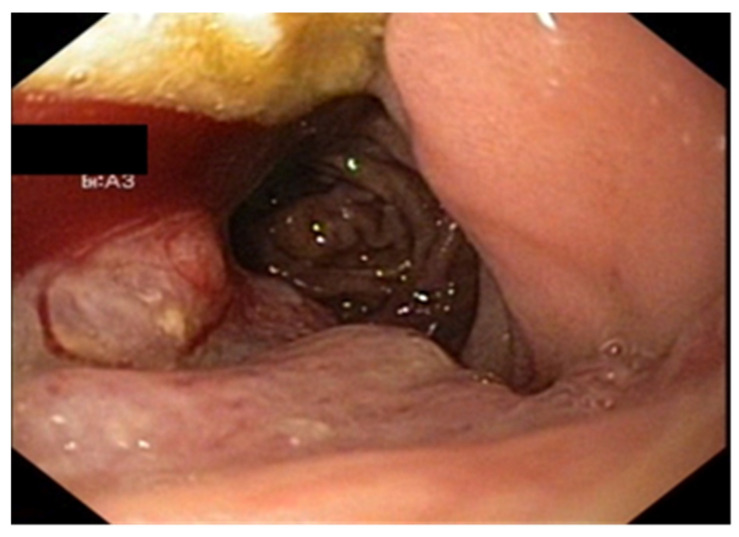
Upper digestive endoscopy: visible carcinoma located on the second part of the duodenum.

CT scans represent the most used investigation for the diagnosis of SBT (60%). In 81.3% of cases, the tumor was visible at the CT scan (Figure 2, Figure 3, Figure 4 and Figure 5); the mean diameter was 59.8 mm. The presence of lymph nodes was noted in one-third of malignant SBT cases, while metastases were present in 25.6%. Upper digestive endoscopy was performed in 34 cases (Figure 6 and Figure 7), with endoscopic resection in 6 cases; lower digestive diagnostic endoscopy was used in 12 cases. Transabdominal ultrasound was used in 21 cases; however, it detected SBT in only 19%. Videocapsule endoscopy was performed in only four cases, all with a negative CT scan and endoscopy.

Of 58 malignant tumors, 89.7% were primitive and 10.3% were metastases. Carcinoma represented the most frequent primitive malignant SBT (48.3%), with adenocarcinoma being the most encountered subtype, although we recorded two cases of squamous cell carcinomas of the small bowel. Lymphoma was the second primitive malignant SBT (13.8%), followed in equal proportion by neuroendocrine tumors and GISTs (10.3%).

### 3.2. Treatment Particularities

**Figure 8:**
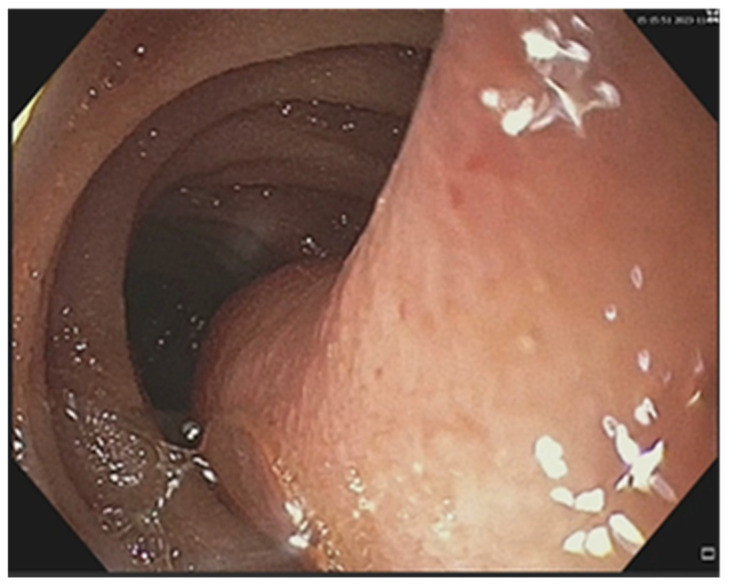
Upper digestive endoscopy: pedunculated lipoma of the third part of the duodenum.

**Figure 9:**
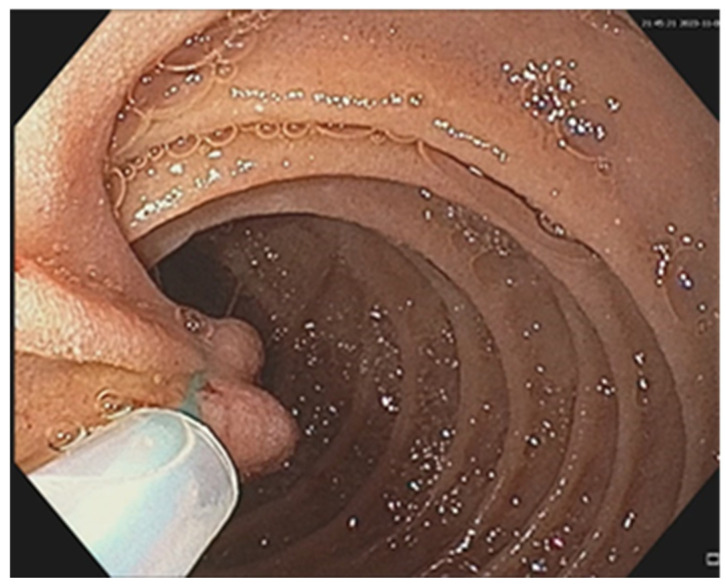
Upper digestive endoscopy: same duodenal lipoma and the placement of an endoloop to the pedunculum, followed by resection.

**Figure 10:**
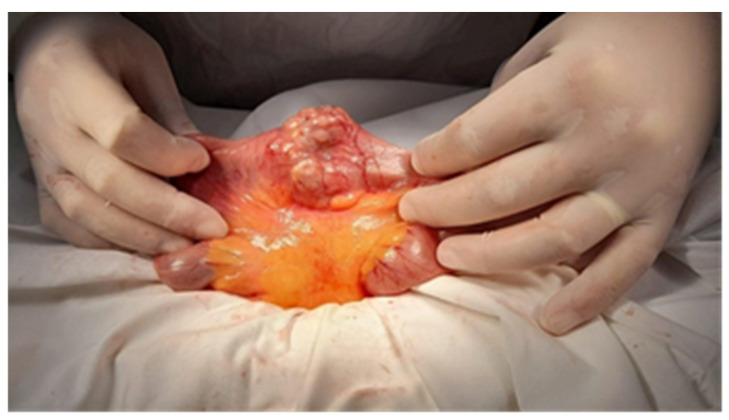
Surgical intraoperative aspect: jejunal metastasis. The pathology exam revealed an ileal lymphoma.

**Figure 11:**
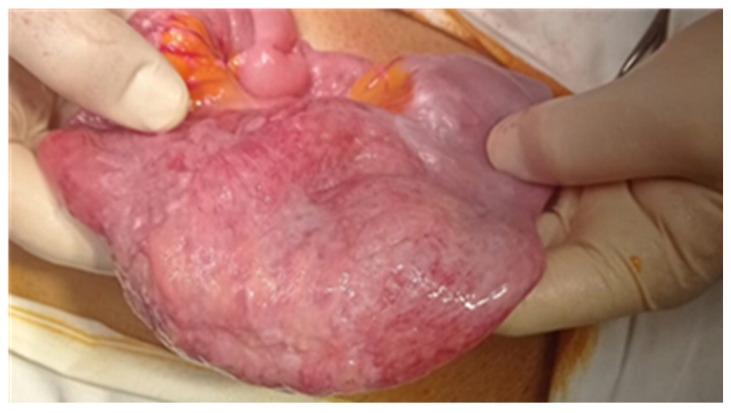
Surgical intraoperative aspect of an ileal tumor.

**Figure 12:**
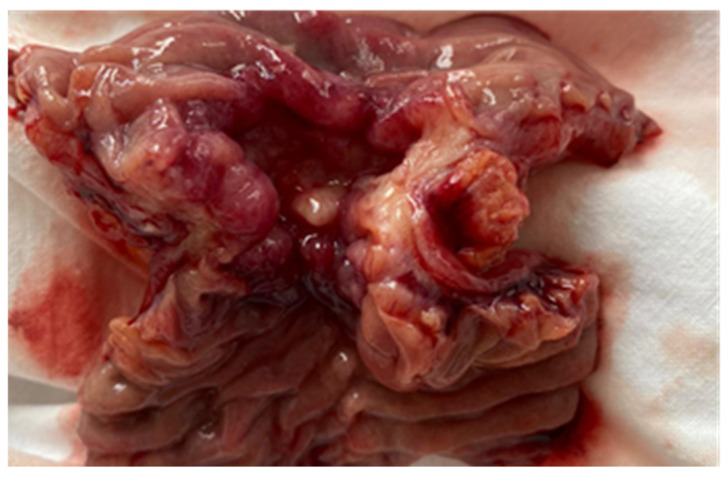
The surgical intraoperative aspect of a jejunal stenosing tumor. Pathology examination revealed an adenocarcinoma.

**Figure 13:**
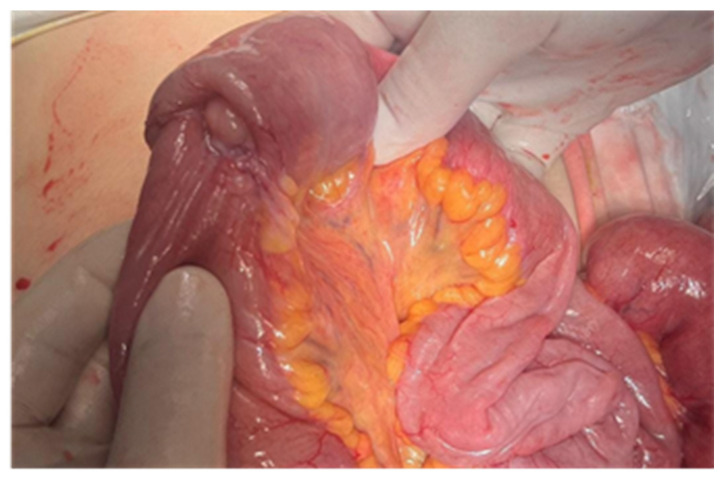
Surgical intraoperative aspect of jejunal tumor complicated with invagination. Pathology examination has shown a carcinosarcoma.

Endoscopic resections was performed in 7 cases of benign tumors (Figure 8 and Figure 9), while surgical resection was performed in 47 cases (Figure 10, Figure 11, Figure 12 and Figure 13). Internal or external derivations were needed in 10 cases; laparotomy with biopsy for confirmation was performed in 16 cases.

### 3.3. Immunohistochemistry

**Figure 14:**
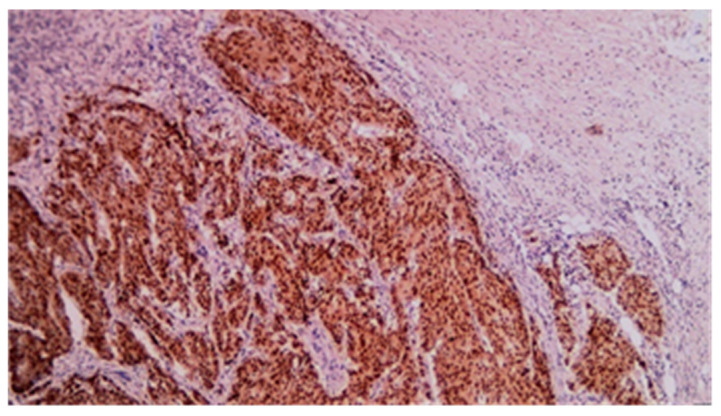
CDX2 immunohistochemical staining of mixed adenocarcinoma–squamous carcinoma of the small bowel (20×).

**Figure 15:**
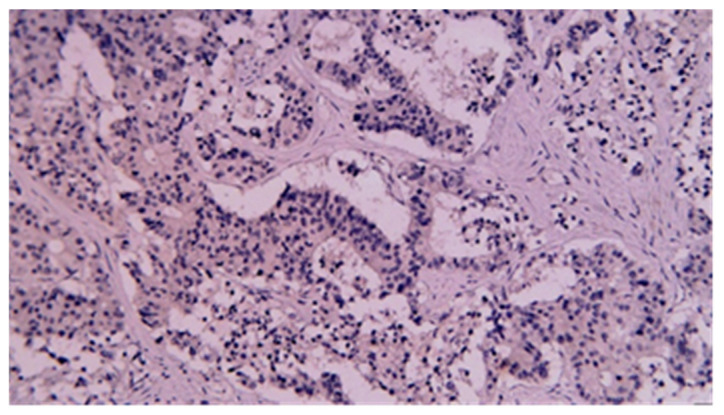
P63 immunohistochemical staining of mixed adenocarcinoma–squamous carcinoma of the small bowel (20×).

**Figure 16:**
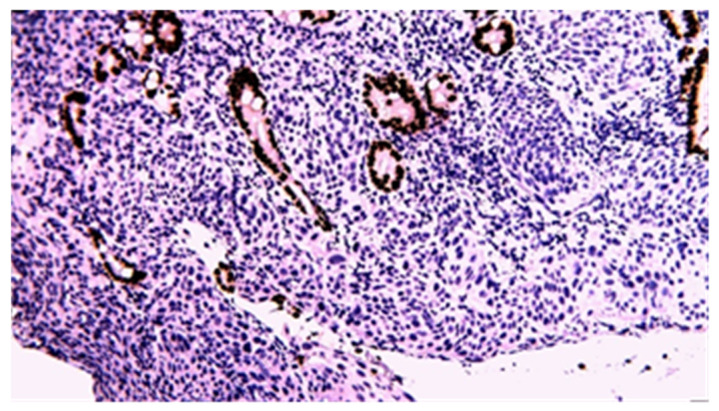
CDX2 immunohistochemical staining of squamous carcinoma of the small bowel (20×).

**Figure 17:**
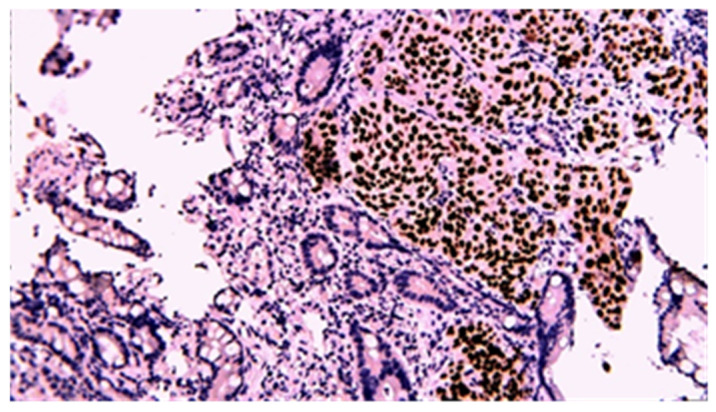
P63 immunohistochemical staining of squamous cell carcinoma of the small bowel (20×).

**Figure 18:**
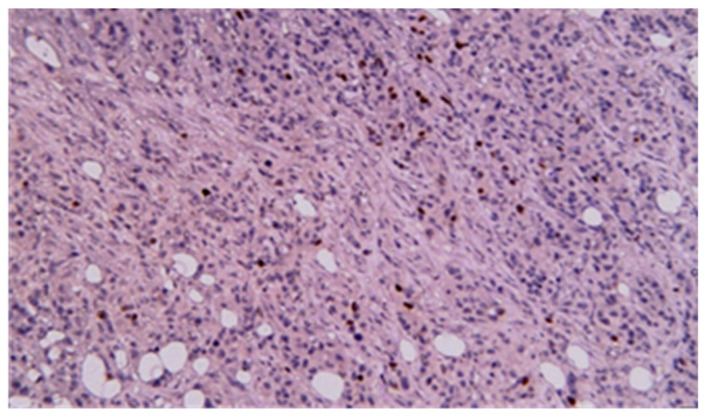
CDX2 immunohistochemical staining of poorly differentiated carcinoma of the small bowel (20×).

**Figure 19:**
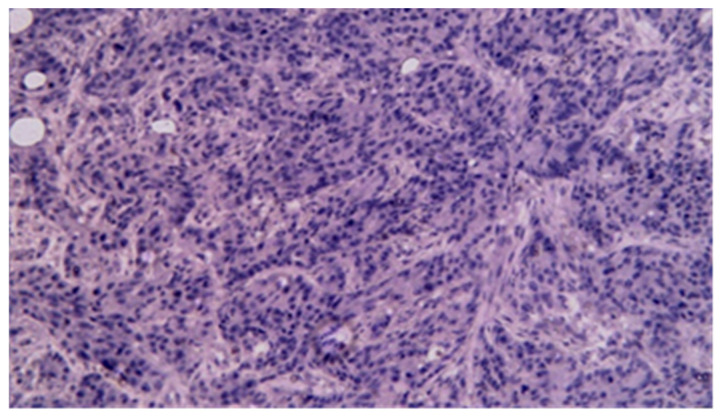
P63 immunohistochemical staining of poorly differentiated carcinoma of the small bowel (40×).

Immunohistochemistry was performed in all gastrointestinal stromal tumors and revealed the presence of CD117, CD34, and DOG1 in all cases; alpha-actin was present as a vascular marker in one case and had a zonal distribution in another. Ki-67 was used for GIST classification according to the criterion of malignant potential. The diagnosis of intestinal lymphomas was confirmed by immunohistochemistry in dedicated hematological centers. For carcinomas, immunohistochemistry was performed in seven cases; two adenocarcinomas had both CD19 and CA 19-9 positivity, with CD10, S100, P53, CAIX positivity, and vimentin, tyrosine-kinase, and CD56 negativity in one case. CDX2 positivity was noted in all adenocarcinomas, while P63 positivity was noted in all squamous cell carcinomas and focally positive in adenocarcinomas with squamous differentiation (Figure 14, Figure 15, Figure 16, Figure 17, Figure 18 and Figure 19).

Of 28 patients with small bowel carcinoma, 3 died soon after surgery; 14 of 23 remaining patients had postoperative chemotherapy, and none had preoperative chemotherapy or radiotherapy. We had no therapeutic data regarding the other two cases of carcinoma, as well as for lymphomas, NETs, GISTs, and sarcomas.

### 3.4. Survival Analysis

**Figure 20:**
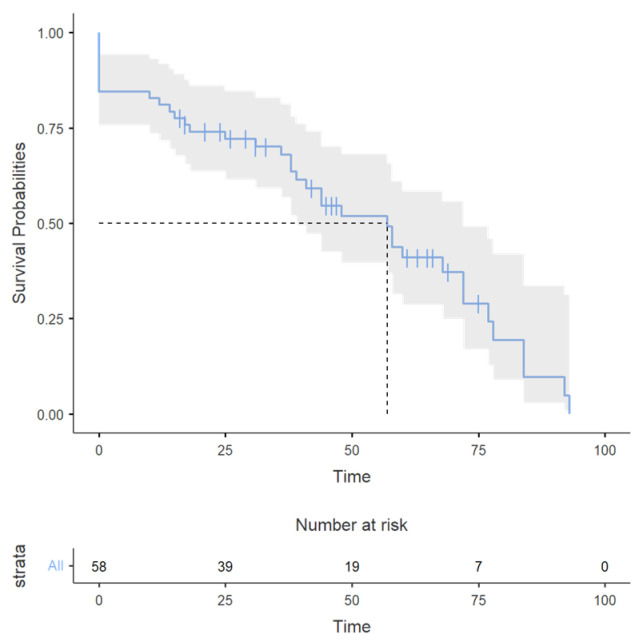
The Kaplan–Meier curve for all SBT; the *X*-axis represents the time (months).

**Figure 21:**
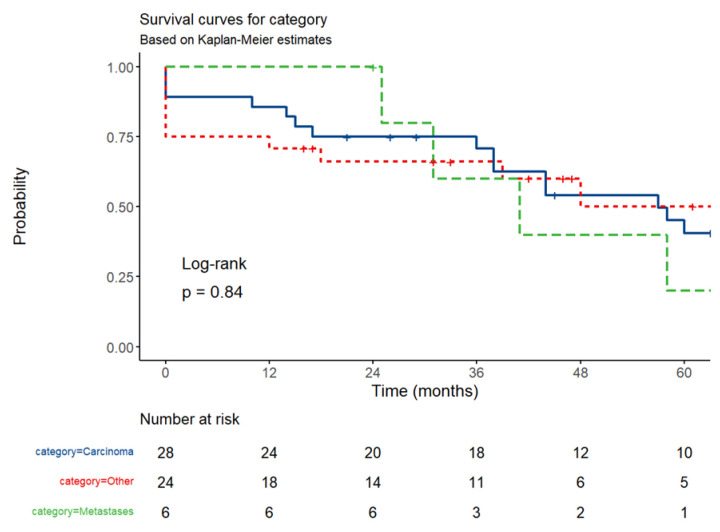
The Kaplan–Meier curve for carcinomas, SB metastases, and other tumors (NET, lymphoma, sarcoma, GIST). The *X*-axis represents the time (months).

Because carcinomas represented the most frequent malignant SBT, we evaluated the prognosis for all malignant SBT and among the three histological categories of small bowel tumors (carcinoma, metastases, and other tumors including lymphoma, sarcoma, GIST, and NET) by constructing the Kaplan–Meier curve (Figure 20 and Figure 21).

The number of survivors decreased over time in all categories (Figure 21); the decline was more gradual for carcinomas, steeper in later time points for NET, GISTs, lymphoma, and sarcoma, and faster after the third time-point for small bowel metastases. Despite these trends, the survival curves remained statistically indistinguishable. For carcinoma patients, 12-month survival was 86% (95%CI, 73.69–99.71%), 36-month survival was 71% (55.64–90.18%), and 60-month survival was 41% (25.03–65.93%). For patients with small bowel metastases, 12-month survival was 100%, 36-month survival was 60% (29.33–100%), and 60-month survival was 20% (3.46–100%). For the group of patients with lymphoma, sarcoma, NET, and GISTs, 12-month survival was 71% (54.80–91.57%), 36-month survival was 66% (49.46–88.37%), and 60-month survival was 50% (30.46–82.34%). The log-rank test revealed no statistically significant difference in survival between the groups (*p* = 0.84), possibly because of the small number of patients. Compared to carcinoma patients, patients with lymphomas, GISTs, and neuroendocrine tumors had a hazard ratio (HR) of 0.96 (95% CI: 0.47–1.93; *p* = 0.902), and those with small bowel metastases from other tumors had an HR of 1.29 (95% CI: 0.43–3.85; *p* = 0.648) relative to carcinoma patients, indicating no significant difference in risk.

After stratifying survival by tumor type, subgroup analysis revealed significant heterogeneity in median survival across tumor types. SARs had a numerically higher median survival (72 months) but with a CI spanning 0–NA, implying potential outliers or small sample size. Carcinoma had a median survival (57 months, 95% CI: 38–78). GIST and lymphoma showed intermediate outcomes (both 48 months), although lymphoma’s wide CI (0–NA) suggests uncertainty due to limited events. Small bowel metastases (41 months, CI: 31–NA) and NET (39 months, CI: 18–NA) had the poorest survival, with lower bounds of CIs indicating early risk. The median survival was 89 months for stage II carcinoma, 58 months for stage III, and 38 months for stage IV.

## 4. Discussion

In our study, malignant tumors represent 75% of the cases; primitive carcinomas were the most frequent malignant tumors (48.1%), followed by lymphomas, neuroendocrine tumors, stromal tumors, and six cases of metastatic small bowel tumors (three from melanoma, two from urothelial tumor, and one from pulmonary carcinoma). Most primitive carcinomas were adenocarcinomas (24/28 cases). In the literature, adenocarcinoma was the most frequent malignant SBT in most studies [2,5,10,69,70,71], whereas in other studies, small bowel stromal tumors were the most frequent [2,5,65], while lymphoma was almost equal to carcinoma in two studies [26,72]. Most of the small bowel tumors included were malignant, similar to the literature data [2,5,37,49,65,66]; some studies based on balloon-assisted enteroscopy have, however, shown a predominance of benign tumors [55]. Detailed data are provided in Supplementary Table S1.

The mean age was similar for benign and malignant tumors (63.1 for benign lesions and 64 years for malignant tumors, respectively); ages as young as 47 for benign tumors and 35 for malignant tumors were noted. The age distribution was also influenced by excluding pediatric patients, because cases of SBT, almost all benign, can also be encountered in patients under 18 years. Male gender was predominant in both benign (61.9%) and malignant small bowel tumors (62.3%). Most published studies have also noted a male predisposition for small bowel tumors [37,38,39,40,42,55], but others have not confirmed this [7,25,27,72].

Most patients with SBT have undergone radical or palliative surgery; endoscopic resection was performed in 7 cases. Perioperative death was noted in 10 cases (12.5%), 7 cases being associated with complications (4 with intestinal occlusion and 3 with severe bleeding), and the other 3 cases had advanced, unresectable malignant tumors. The most frequent causes for the lack of surgery were consent denial by the patient (10 cases), advanced diseases (8 cases, 7 with distant metastases and 1 with locally advanced disease), 2 cases with a diagnosis of lymphoma, and 1 case with acute cerebrovascular disease. Due to the presence of nonspecific symptoms, the diagnosis was frequently made in the advanced stages. An abdominal CT scan represents the main imaging method used for diagnosis in our patients, with a high detection rate (81.3%); most tumors were large at the moment of diagnosis, with only five cases having tumors between 20 and 30 mm. Upper and lower digestive endoscopy proved useful for tumors located in the duodenum and terminal ileum, respectively.

In our study, the survival of patients with small bowel malignant tumors and also of those with small bowel carcinoma was low, with a 41% 5-year overall survival rate (between 20 and 50% according to the tumor type). In the literature, 5-year survival rates of 20–30% are reported [1,6,26,73,74,75], but rates as low as 9% [76] and as high as 57.2% [70] have also been reported. In Romania, a study on operated patients with small bowel primitive carcinomas and 25 months’ median surveillance has shown a median survival of 13 months, and a 5-year survival of 30%, with an insufficient number of cases for statistically accurate differentiation between patients who received radical surgery and those with palliative treatment [10]. The mean overall survival and 5-year overall survival were lower in adenocarcinoma compared to NET and GIST [2,6,7,26,70]. The median overall survival in our study was 57 months for small bowel carcinomas, 48 months for GISTs and lymphomas, 72 months for sarcomas, and 57 months for all malignant SBT. The low 5-year survival rate in our study may be explained by the high rate of advanced disease (9/26 carcinomas, 3/6 NETs, 1/3 sarcomas, and 1/6 GISTs were stage IV tumors, while six cases were metastatic SBTs). Advanced small bowel carcinoma is associated with a dismal prognosis, with a median overall survival of 13.8 months and a 14.7% one-year survival rate [77].

Out of 28 patients with carcinoma, 2 had a primary squamous cell carcinoma of the small bowel (one being purely squamous cell carcinoma and the other having a mixed adenocarcinoma–squamous cell carcinoma type); another metastasis originated from a urothelial squamous cell carcinoma. Primitive squamous cell carcinomas of the small bowel are rarely encountered; a 2016 literature review found 22 cases [15]. WHO defined adenosquamous small bowel tumors as tumors including at least 10% adenocarcinoma and squamous cell carcinoma components [22]. Several possible patterns are described in the literature: aberrant differentiation of pluripotent stem cells to malignant squamous cells, the previous existence of a local ectopic squamous epithelium or squamous metaplasia with subsequent malignant transformation favored by chronic mucosal lesions [15,17,22,23]. Collision tumors or partial transformation from adenocarcinoma may explain the appearance of adenosquamous carcinoma [22]. It is difficult to differentiate between primary or metastatic squamous cell small bowel tumors. There are several criteria such as apparent atypia and nested distribution, the formation of a keratinized pearl, the lack of glandular components and glandular epithelium, and no evidence of involvement of primary SCC of other organs [17,18]. The presence of squamous metaplasia of the glandular epithelium favors the primary small bowel tumor if most of the cancer cells are deep in the wall of the SI, whereas in cases with little involvement of the mucosa, the metastatic nature of the lesion is more likely, especially if tumoral cells reach mucosal surfaces [18]. Both our patients were operated on and survived more than 5 years (one died at 78 months and another is still alive at 63 months); neither patient has followed postoperative chemotherapy. Clinicopathological characteristics, standardized therapy, and survival were hard to evaluate because of the rarity of the cases [15,22]; in one study, 66.7% of the squamous SB carcinomas died within one year after diagnosis (4/9 being metastatic tumors) [22], whereas in another review, from 22 patients, 16 cases were operated on, 4 and 3 were treated by chemotherapy and radiotherapy, and 54.5% died during follow-up [15].

There are several limitations to our study. Because of the rarity of SBT, our patient group was relatively small, which partially impaired the statistical analysis; the diverse type of tumors (benign or malignant, primitive or metastatic, carcinoma, lymphoma, sarcoma, NET, or GIST) further fragmented the patient groups and consequently impacted the statistical power of our analysis. While being difficult to conduct, a multicentric study may overcome the issues related to the small size; an extended timeframe may also be desired. However, changes in diagnostic and therapeutic modalities may render the study unsound for survival analysis. Two pediatric cases were excluded, both with benign tumors; most small bowel tumors in children are benign (infantile polyps, hamartoma, Peutz–Jeghers polyposis). Data related to lymphoma treatment were not available. Long-term survival (five years after the diagnosis) was impossible for patients admitted in the last three years of our study.

## 5. Conclusions

We diagnosed 80 small bowel tumors during the 7-year study period; 72.5% of small bowel tumors were malignant, the most frequent carcinoma, followed by lymphoma, GIST, and neuroendocrine tumors. Two squamous cell carcinomas were also noted. CT scans and upper digestive endoscopy were the main imaging investigations for the diagnosis. We found that 58.8% of tumors were resected surgically, and 9% by endoscopy. Survival was poor, with 5-year rates of 41% for carcinomas. Despite technical progress regarding diagnosis by videocapsule endoscopy, CT scan, and MRI, the early diagnosis and treatment of small bowel tumors is still difficult to achieve because of their nonspecific symptoms and the limitations of imaging methods for the diagnosis.

## Figures and Tables

**Table 1:** Baseline characteristics of the studied cohort.

	No (%)
**Year of diagnosis**	
2017/2018/2019/2020/2021/2022/2023	6/13/11/5/14/11/20
**Age** mean ± SD (min-max)	
Benign	63.1 ± 9.4 (47–78)
Malignant	64 ± 11.6 (34–85)
**M/F** (%)	
Benign	14/8 (63.6)
Malignant	36/22 (62.1)
**Complains**	
**Site**	
Duodenum	28 (35)
Jejunum	21 (26.4)
Jejuno-ileum	3 (3.8)
Ileum	23 (28.8)
Multiple	5 (6.4)
**Symptoms at admission**	
Abdominal pain	43 (53.8)
Nausea, vomiting	25 (31.3)
Constipation/diarrhea	13/1 (16.3/1.2)
Bleeding	12 (15)
Anemia	8 (10)
Weight loss	3 (3.8)
Fatigue	4 (5)
No symptoms	6 (7.5)
**Complications**	
Occlusion	12 (15)
Invagination	6 (7.5)
Perforation	4 (5)
**CT scan** number	48 (60)
Tumor (%)	39 (81.3)
Lymph nodes (% from malignant tumors)	12 (33.3)
Metastasis (% from malignant tumors)	22 (37.9)
**Transabdominal ultrasound** [No (%)]	21 (26.3)
Tumor	4 (19)
**Endoscopy**	
Upper digestive endoscopy	34 (42.5)
Lower digestive endoscopy	12 (15)
**VCE**	4 (5)
**Surgery**	52 (62.6)
Radical	47 (58.8)
Palliative	10 (12.5)
Laparoscopy with biopsy	16 (20)
**Endoscopic resection**	7 (8.8)
**Pathology** No (%)	
Benign	22 (27.5)
GIST	8 (36.4)
Fibroid inflammatory	6 (27.5)
Epithelial (adenoma, hyperplastic)	5 (22.7)
Lipoma	1 (4.5)
Hemangioma	1 (4.5)
Hamartoma	1 (4.5)
Malignant	58 (72.5)
Primitive carcinoma	28 (48.3)
Adenocarcinoma	24 (41.4)
Squamous cell carcinoma (1 mixed)	2 (3.4)
Undifferentiated carcinoma	1 (1.7)
Carcinosarcoma	1 (1.7)
Lymphoma	8 (13.8)
NET	6 (10.3)
GIST	6 (10.3)
Metastasis	6 (10.3)
Carcinoma (1 from squamous cell carcinoma)	3 (5.2)
Melanoma	3 (5.2)
Grading (primary malignant SBT)	
G2	11
G3	6
G4	1
Staging (primary malignant SBT)	
pT3	7
pT4	5
pN0	5
pN1	4
pN2	1
M1/M0	14/22
Invasion (primary malignant SBT)	
Vascular	6
Lymphatic	3
Perineural	6
In-hospital death [No (%)]	12 (10)

## Data Availability

The data presented in this study are available on request from the corresponding author.

## Supplementary Materials

The following supporting information can be downloaded at: https://www.mdpi.com/article/10.3390/cancers17091465/s1, Table S1: Studies describing small bowel tumors.
