# Supplementary material for: Small Bowel Tumors: A 7-Year Study in a Tertiary Care Hospital
Source: Cancers (Basel). 2025 Apr 27;17(9):1465. doi: 10.3390/cancers17091465 (PMC12071178; doi:10.3390/cancers17091465)
Supplement: Supplementary file 1 [file cancers-17-01465-s001.zip › cancers-3547768-supplementary.pdf]

Author	Country	No. of cases	Timeframe	Inclusion criteria	Type of malignant tumors	Reference
Kim CH et al	Korea	81 (76 malignant)	2007-2019	All surgical benign and malignant primary SBT	49.4% GIST, 32.1% lymphomas, and 6.2% adenocarcinomas.	2
Sahin E et al	Turkey	37 (27 malignant)	2000-2023	All surgical benign and malignant SBT	23.8% GIST, 19% adenocarcinoma, 16.7% lymphoma; 21.4% metastasis, and 2.4% NET.	5
Obleaga SV et al	Romania	46 (46 malignant)	2014-2024	Surgical cases, primitive malignant SBT	56.5% adenocarcinomas, 23.9% lymphomas, 17.4% GIST, and 2.2% NET.	10
Farhat MH et al	Liban	33 (33 malignant)	1986–2006	All admitted malignant primary and metastatic SBT	Lymphoma 36.4%, adenocarcinoma 33.3%, GIST 12.1%, and NET 3%.	26
Dolu S et al	Turkey	90 (53 malignant)	2006-2020	All benign and malignant SBT diagnosed by double-balloon enteroscopy	40.6% adenocarcinoma, 37.5% GIST.	37
Vere CC et al	Romania	11 (6 malignant)		Videocapsule based. All benign and malignant SBT	3 GIST, one adenocarcinoma, and one neuroendocrine tumor diagnosed. An ampullary malignant tumor was also diagnosed.	49
Han JW et al	Korea	79 (16 malignant)	2004-2012	Al benign and malignant SBT diagnosed by surgery/double-balloon enteroscopy	7 adenocarcinomas, 5 GIST, 3 lymphomas, and one NET diagnosed.	55
Yoo AI et al	Korea	28 (28 malignant)	2010-2018	Al admitted malignant SBT diagnosed by VCE/balloon enteroscopy	8 patients had lymphoma, 8 had primary adenocarcinoma, 7 had GIST, and 5 had metastasis.	66
Sánchez-Ramón A et al	Mexico	38 (38 malignant)	1990-2011	All admitted malignant SBT	36.4% adenocarcinomas, 26.3% neuroendocrine tumors, 21.1% sarcomas, and 10.5% lymphomas diagnosed.	67
Zhang S et al	China	456 (418 malignant)	1999-2016	Surgical treated benign and malignant SBT	52.9% adenocarcinoma, 33.6% GIST, and 3.5% NET	68
Rondonotti E et al	Europe (29 centers from 10 European countries)	128 (128 malignant)	VCE first use-2006	Videocapsule-based. All malignant SBT	10% multiple tumors, 10% metastasis, 20% adenocarcinoma, 32% GIST.	69
Margaritescu ND et al	Romania	31 (18 malignant)	2002-2013	Surgical benign and malignant SBT	38.9% GIST, 22.2% adenocarcinomas, 16.7%, and 11.1% each for lymphomas and NET.	71
Tarcoveanu	Romania	63 (45 malignant)	1992-2010	Surgical benign and malignant SBT	53.3% adenocarcinoma, 22.2% lymphoma, 11.1% GIST, 9% metastases, 2.2% sarcoma, 2.2% NET	72
Negoi I et al	Romania	57 (57 malignant)	2000-2015	Surgical cases, primitive malignant SBT	GIST 42.1%, adenocarcinoma 33.3%, lymphoma 14%, and NET 3.5%.	73

Table S1
